# GMI, an Immunomodulatory Peptide from *Ganoderma microsporum*, Restrains Periprosthetic Joint Infections via Modulating the Functions of Myeloid-Derived Suppressor Cells and Effector T Cells

**DOI:** 10.3390/ijms22136854

**Published:** 2021-06-25

**Authors:** Kuo-Ti Peng, Jiun-Liang Chen, Liang-Tseng Kuo, Pei-An Yu, Wei-Hsiu Hsu, Chiang-Wen Lee, Pey-Jium Chang, Tsung-Yu Huang

**Affiliations:** 1 Department of Orthopedic Surgery, Chang Gung Memorial Hospital, Puzi 61363, Taiwan; yq0139@cgmh.org.tw (J.-L.C.); light71829@gmail.com (L.-T.K.); b9002065@cgmh.org.tw (P.-A.Y.); 7572@cgmh.org.tw (W.-H.H.); 2 College of Medicine, Chang Gung University, Taoyuan 33303, Taiwan; 3 Division of Basic Medical Sciences and Recurrent Diseases, Health Promotion Research Center, Department of Nursing, Chang Gung University of Science and Technology, Puzi 61363, Taiwan; cwlee@mail.cgust.edu.tw; 4 Research Center for Industry of Human Ecology, Research Center for Chinese Herbal Medicine, Chang Gung University of Science and Technology, Taoyuan 33303, Taiwan; 5 Department of Rehabilitation, Chang Gung Memorial Hospital, Puzi 61363, Taiwan; 6 Graduate Institute of Clinical Medical Sciences, College of Medicine, Chang-Gung University, Taoyuan 33302, Taiwan; peyjiumc@mail.cgu.edu.tw; 7 Department of Nephrology, Chang-Gung Memorial Hospital, Chiayi 61363, Taiwan; 8 Division of Infectious Diseases, Department of Internal Medicine, Chang Gung Memorial Hospital, Chiayi 61363, Taiwan; 9 Department of Nursing, Chiayi Campus, Chang Gung University of Science and Technology, Chiayi 61363, Taiwan

**Keywords:** periprosthetic joint infections, GMI, T cells, myeloid-derived suppressor cells

## Abstract

Periprosthetic joint infections (PJIs) caused by *Staphylococcus aureus* infection are difficult to treat due to antibiotic resistance. It is known that the biofilms from methicillin-resistant *S. aureus* (MRSA) promote expansion of myeloid-derived suppressor cells (MDSCs) to suppress T-cell proliferation and benefit bacterial infections. This study finds that GMI, a fungal immunomodulatory peptide isolated from *Ganoderma microsporum*, suppresses MDSC expansion to promote the proliferation of cytotoxic T cells. The enhancement is likely attributed to increased expression of IL-6 and TNF-α and reduction in ROS expression. Similar beneficial effects of GMI on the suppression of MDSC expansion and IL-6 expression are also observed in the whole blood and reduces the accumulation of MDSCs in the infected bone region in a mouse PJI infection model. This study shows that GMI is potentially useful for treating *S. aureus*-induced PJIs.

## 1. Introduction

*Staphylococcus aureus* is a Gram-positive bacterium causing periprosthetic joint infections (PJIs) [1,2]. In primary total joint arthroplasties, the PJI incidence is 1–2%, although 2–6% was observed in revision joint arthroplasties [3]. Although many strategies have been developed to improve PJI treatments [4,5], including debridement with prosthesis retention, one-stage or two-stage reimplantation, and antibiotic therapy, high treatment failure rates of 10-30% during follow-ups [1,6,7,8] cause a huge financial burden on patients [9,10]. PJI caused by *S. aureus* infection is particularly difficult to treat as this pathogen produces biofilms and is highly resistant to many antibiotics [11,12,13,14,15,16]. Furthermore, *S. aureus* often contaminates surgical instruments and implants, making the treatment of PJI challenging [17,18]. Therefore, this study seeks a treatment method that improves antibiotic therapy of PJIs caused by MRSA infection.

Myeloid-derived suppressor cells (MDSCs), an innate immune cell subset, are known to decrease immune-mediated pathology of the hosts to prevent the collateral damage caused by host immune responses [19]. However, the decreased immunity often provides advantages to bacterial infection [19]. For instance, *S. aureus* promotes the expansion of MDSCs from bone marrow cells (BMCs) to suppress T-cell proliferation, resulting in decreased host immunity [20]. Therefore, compounds that prevent or reverse the expansion of MDSCs may be beneficial to the treatments of PJI.

Many natural compounds have been reported to have immunomodulatory and overall health-benefiting effects to people. These compounds are considered safer and less toxic than the synthetic drugs [21,22]. Fungal immunomodulatory proteins (FIPs) are a group of peptides, which are known to modulate immunity [23,24]. So far, 38 types of FIPs have been identified, which are classified into five subgroups [24]. It is well known that FIPs have anti-inflammatory, anti-cancer, and anti-allergic activities. FIPs also inhibit replication of respiratory syncytial virus and microglia [23,24,25,26]. *Ganoderma* spp., also called Lingzhi or Reishi, is a popular chemoprevention mushroom. GMI (GenBank AGU04723.1), the FIP from *Ganoderma microsporum*, is a 111-amino acid peptide and one of the active components in the mushroom. The gene encoding GMI, *Pichia pastoris,* has been cloned and can be produced by yeast and is widely used in biochemical research and biotech industries [24,27]. The recombinant GMI was demonstrated to regulate cytokine expression, inhibit lung cancer cells [28,29,30,31], and have anti-inflammatory and neuroprotective activities [26]. This study finds that both in vitro and in an *S. aureus*-induced mouse PJI model, GMI promotes T cell proliferation via suppression of MDSC expansion, increases cytokine expression, and reduces ROS, showing that GMI is an attractive candidate for PJI treatment.

## 2. Materials and Methods

### 2.1. Animals

Male C57BL/6J mice that were 8–12 weeks old with weights from 22–25 g were purchased from National Laboratory Animal Center (Taiwan). The mice were acclimated to a room with controlled temperature (25 °C) and humidity (50 ± 10%) with a 12-h day-night cycle. The studies were approved by the Institutional Animal Care and Use Committee of the Chang Gung Memorial Hospital (IACUC permit number: 2019122309) and were performed in accordance with the Animal Protection Law by the Council of Agriculture, Executive Yuan (Taiwan) and the guidelines of National Research Council (USA) for the care and use of laboratory animals.

### 2.2. GMI, S. aureus and Biofilm Preparation

GMI is a fungal immunomodulatory protein from *Ganoderma microsporum* (NCBI protein ID AGU04723.1) and was provided by MycoMagic Biotechnology Co, Ltd. (Taipei, Taiwan).

*S. aureus* ATCC43300, a MRSA strain, was purchased from the Bioresource Collection and Research Center (BCRC, Hsinchu, Taiwan). The bacteria were cultured in brain heart infusion (BHI) media (Bacto, Sydney, Australia). After culturing for 4 days, *S. aureus* biofilms were then prepared by autoclaving and centrifugation according to a method reported elsewhere [20]. The biofilms were suspended in RPMI 1640 medium; the number of proteins in the biofilms was quantified using a BCA protein assay kit (Pierce) and was adjusted to 0.2 mg/mL with RPMI 1640 medium. For experiments using live bacteria, *S. aureus* was harvested by centrifugation and enumerated spectrophotometrically by measuring the turbidity at 600 nm (A_600_) and by viable cell counts.

### 2.3. Isolating MDSCs from Mouse Bone Marrow by Cell Sorting

Bone marrow cells (BMCs) in mouse femur were collected and treated with anti-CD11b antibody conjugated with FITC and anti-Gr1 antibody conjugated with PE according to the methods described earlier [20]. MDSCs, which were stained by both antibodies, were sorted with a FACSAria Fusion cell sorter (BD). The purity of the isolated cell population was then verified by flow cytometry.

### 2.4. Analysis of MDSC Expansion

Transwell plates with 0.4-μm pore size inserts were used to examine how biofilms and GMI influenced MDSC expansion. BMCs were cultured in the lower compartment in RPMI 1640 medium containing 10% fetal calf serum with different concentrations of GMI; *S. aureus* biofilms (0.2 mg/mL) were added to the inserts. The membrane in the inserts prevented the presence of biofilm debris in the lower compartment to interfere with flow cytometry analysis of BMCs and MDSCs. After culturing for 48 h, cells stained with anti-CD11b-FITC and anti-Gr1-PE antibodies were analyzed using flow cytometry (BD FACSCanto II) to determine the numbers of MDSC proportion. Acquisition of flow cytometry data from the flow cytometer was performed using FACSDiva software (BD Biosciences, Franklin Lakes, NJ, USA). The number of events analyzed was 10,000 per sample. Analysis was performed using FlowJo software (Tree Star, Ashland, OR, USA).

### 2.5. Analysis of Tc, T_H_, Treg Cells by Flow Cytometry

After treating BMCs with biofilms for 48 h, MDSCs were isolated by sorting as described above. MDSCs (1 × 10^5^ cells) were then treated with GMI, washed with PBS, and cocultured for 48 h with CFSE-labeled spleen T cells (2 × 10^5^ cells) that were stimulated by Dynabeads Mouse T-Activator CD3/CD28 (Gibco). The mouse spleen T cells were purified and labeled with CFSE according to a method described elsewhere [20]. Cells were then stained with anti-CD3-PerCP-Cy5.5 (BD Bioscience), anti-CD4-APC-H7 (BD Bioscience), anti-CD8-PE-Cy7 (BD Bioscience), anti- CD25-PE (BD Bioscience), and anti-FoxP3-Alexa Fluor 647 (BD Bioscience) to analyze Tc, T_H_, and Treg cells by flow cytometry.

### 2.6. Preparation of Blood Samples for Flow Cytometry Analysis

For determining the effect of GMI on the dynamic changes of the immune cell populations in the peripheral blood of mice, 100 μL blood samples were collected. After lysing red blood cells with ACK Lysing buffer (Thermo Fisher, Waltham, MA USA), leukocytes were suspended in PBS containing 2% FBS. T cells were then analyzed by flow cytometry as described above.

### 2.7. Analysis of IL-6 and TNF-α in Culture Medium and Plasma

A Cytometric Bead Array (CBA) Mouse Inflammation kit was used to quantify IL-6 and TNF-α in culture medium and blood. After mixing 50 μL cell culture medium or 50 μL blood with the beads, IL-6 and TNF-α captured by the beads were reacted with mouse inflammation PE detection reagent for two hours and analyzed by flow cytometry.

### 2.8. Measurement of ROS

ROS was measured with 10 µM 2′,7′-dichlorofluorescin diacetate (DCFDA) (Sigma-Aldrich, St. Louis, MO, USA) according to a method described by Evgeniy et al. and analyzed by flow cytometry [32].

### 2.9. GMI Treatment of Mice Infected by S. aureus

Orthopedic implantation was performed in male C57BL/6J mice according to a method described previously [20] and infected with 2 × 10^6^ CFU of *S. aureus* at the intramedullary canal containing the implant. The mice were divided into five groups, including the control group and four different treatment groups (*n* = 4 for each group), which include (i) the group that received GMI (8 mg/kg) only; (ii) the group that was infected with *S. aureus* only; (iii–v) the groups that was injected intraperitoneally with different concentrations of GMI (2 and 8 mg/kg) three times a week after the infection with *S. aureus* for 3 days. Mice that received implants but without *S. aureus* inoculation served as the control-operated group. After injection for 2 weeks, the mice were sacrificed, and the femurs were fixed in 10% formalin.

### 2.10. Fluorescence Molecular Tomography

The PJI mice were divided into four groups, including the control group and three different treatment groups with various concentrations of GMI injection (0, 2, and 8 mg/kg) (*n* = 4 for each group). After GMI injection for 24 h, DilC18 (7)—labeled MDSCs (5 × 10^6^ cells), were injected via tail vein. The infection sites were observed by fluorescence molecular tomography (FMT) at Days 1, 4, and 7. Fluorescence of MDSCs were quantitatively assessed with TrueQuan software.

### 2.11. Statistical Analysis

Results were expressed as mean ± standard deviation (SD) and analyzed statistically by Student’s *t*-test with GraphPad Prism software (GraphPad, San Diego, CA, USA). A *p*-value less than 0.05 was considered significant.

## 3. Results

### 3.1. Influence of GMI on S. aureus Biofilm-Mediated MDSC Expansion

**Figure 1:**
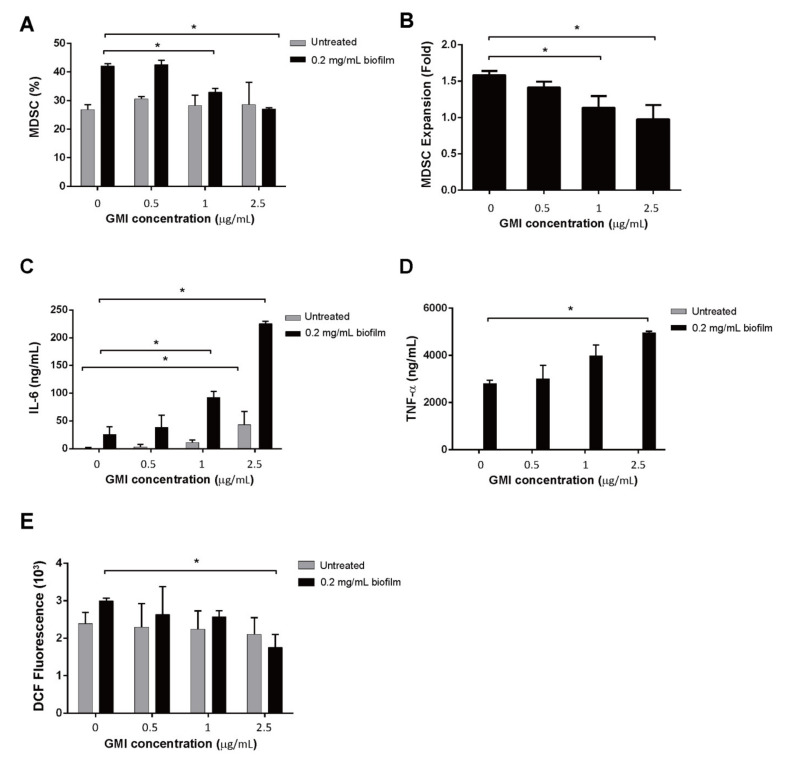
Influence of GMI on MDSC expansion and IL-6, TNF-α, and ROS production. BMCs were treated with *S. aureus* biofilms GMI for 48 h. BMCs were also untreated with the biofilm but treated with GMI for the purpose of comparison. The number of MDSCs, which were CD11b-FITC an Gr1-PE positive, were determined by cell sorting with a cell sorter. The percentage of MDSCs in the whole cell population was presented (**A**). The number of MDSCs from BMCs treated with biofilms was divided by those untreated with biofilms to show the fold increase in the enhancement of MDSC expansion by biofilms (**B**). The amounts of IL-6 and TNF-α produced by the cells were determined using a CBA kit followed by flow cytometry. (**C**,**D**). Cells were treated with 10 µM DCFDA, cell population with ROS production was determined by flow cytometry (**E**). The results were analyzed statistically with Student’s *t*-test. *: *p* < 0.05.

In this study, we investigated whether GMI treatments affected MDSC expansion that was stimulated by *S. aureus* biofilms [20]. We found that in the absence of GMI, 27% of BMCs were expanded to MDSCs (Figure 1A). After treating BMCs with *S. aureus* biofilms, a 1.5-fold increase in MDSC expansion was observed (Figure 1B). The results were consistent with the results of an earlier study which reported that *S. aureus* biofilms activates MDSC proliferation [20]. We also found that although GMI did not seem to promote MDSC expansion from BMCs that were untreated with *S. aureus* biofilms (Figure 1A), GMI treatments impacted the expansion that was stimulated by *S. aureus* biofilms. We found that GMI at 1 and 2.5 µg reduced the expansion from 1.5-fold to 1.2- and 1.1-fold, respectively (Figure 1B).

### 3.2. Activation of IL-6 and TNF-α Expression by GMI

*S. aureus* biofilms are known to stimulate the production of inflammatory cytokines, including IL-6 and TNF-α [33]. In this study, we found that little IL-6 was released into the culture medium by MDSCs that were untreated with biofilms and GMI (Figure 1C). When the cells were treated with 0.5 µg/mL GMI, the amounts of IL-6 increased only a little. However, if the concentration of GMI increased to 1 and 2.5 µg/mL, the amounts of IL-6 increased to 10 ng/mL and 50 ng/mL, respectively (Figure 1C), showing that GMI activates the expression of IL-6 from MDSCs untreated with biofilms. We also found that MDSCs that were treated with biofilms produced 27 ng/mL IL-6, an amount substantially higher than that produced by MDSCs untreated with biofilms (Figure 1C), verifying our earlier findings that *S. aureus* biofilms stimulates IL-6 expression [20]. When biofilm-treated MDSCs were incubated with 0.5 to 2.5 µg/mL GMI, the amounts of IL-6 increased from 25 to 225 ng/mL in a dose-dependent manner (Figure 1C), showing that GMI promotes the expression of IL-6 by the cells treated with biofilms. We also found that MDSCs untreated with biofilms produced little TNF-α even when the cells were incubated with GMI (Figure 1D). However, we found that GMI stimulated TNF-α expression by the cells treated with biofilms. The results showed that the cells untreated with GMI produced 2700 ng/mL TNF-α; the concentration increased from 2800 to 4800 ng/mL in a dose-dependent manner when the cells were treated with 0.5 to 2.5 µg/mL GMI (Figure 1D). These results showed that GMI promotes TNF-α expression by the MDSCs treated with biofilms.

### 3.3. Reduction in ROS Expression by GMI

MDSCs are known to release ROS to suppress T cell responses [34]. We found that although GMI did not seem to affect the release of ROS after the cells were treated with 0.5 and 1 µg GMI (Figure 1E), a reduction in ROS expression by 40% from 3000 to 1800 units DCF fluorescence was observed when compared with the ROS from cells untreated with GMI (Figure 1E). These results showed that GMI reduces the amounts of ROS produced by the cells that are treated with biofilms.

### 3.4. Effects of GMI on the Suppression of T Cell Proliferation by MDSCs

**Figure 2:**
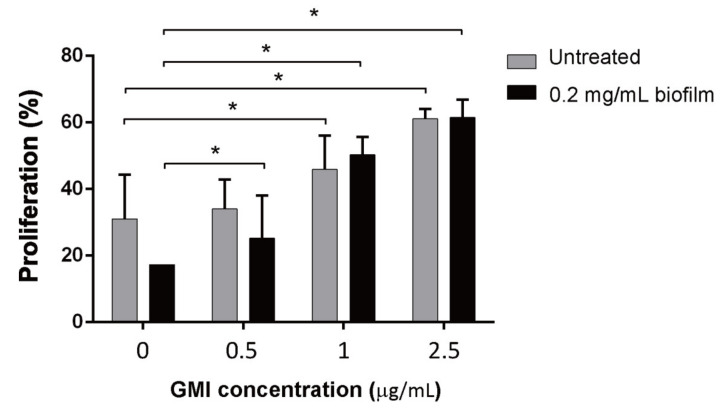
Influence of GMI on the suppression of T cell proliferation by MDSCs. Freshly isolated mouse BMCs were untreated or treated with 0.2 mg/mL *S. aureus* biofilms for 48 h. MDSCs expanded from BMCs were treated with anti-CD11b antibody conjugated with FITC and anti-Gr1 antibody conjugated with PE. The cells that were bound to both antibodies were separated using a cell sorter. MDSCs were treated with different concentrations of GMI for 24 h and then cocultured with activated CFSE-labeled T cells at a ratio of 0.5:1. The results were analyzed statistically with Student’s *t*-test. *: *p* < 0.05.

MDSCs were prepared using a cell sorter from BMCs that were treated or untreated with biofilms. MDSCs were then treated with GMI for 24 h and then cocultured with CFSE-labeled T cells treated with anti-CD3/CD28 antibodies that were conjugated to magnetic beads for 48 h at a ratio of 1:2 (1 × 10^5^:2 × 10^5^) to test how GMI treatment affected MDSC’s ability to suppress T cell proliferation. We found that in the case of MDSCs from BMCs that were untreated with biofilms, the number of T cells increased by 30% if GMI was not added to the culture. The number of T cells increased from 30% to 34%, 46%, 61% after 0.5, 1, and 2.5 µg GMI was added, respectively (Figure 2), showing the promotion of T cell proliferation by GMI. GMI also promoted T cell proliferation that was suppressed by MDSCs from BMCs treated with biofilms. In the absence of GMI, an increase of 20% of T cells was observed (Figure 2), however, the percentage increased to 25%, 50%, 61.5% after 0.5, 1, and 2.5 µg GMI was added, showing that GMI inhibits the suppression of T cell proliferation by MDSCs.

**Figure 3:**
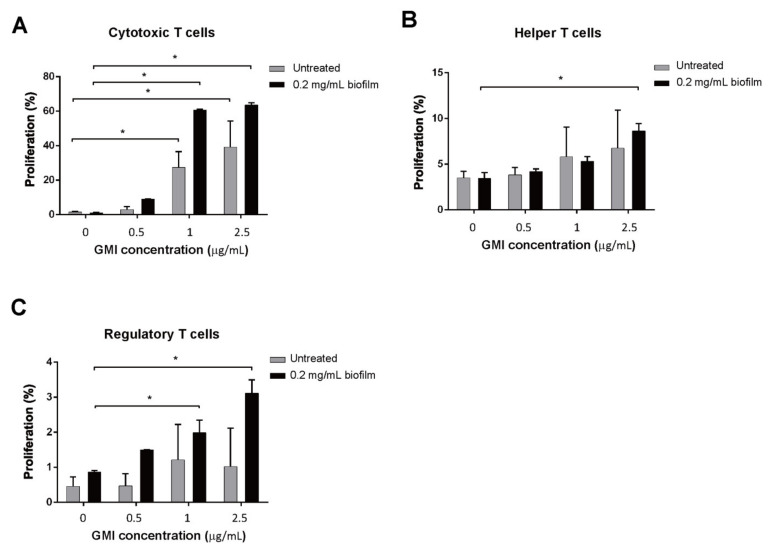
Activation of T cell proliferation that was suppressed by MDSCs. MDSCs that were treated or untreated with biofilm were incubated with GMI. At 24 h after incubation, the cells were cocultured for 48 h with activated CFSE-labeled T cells at a ratio of 0.5:1, and proliferation of Tc (**A**), T_H_ (**B**), and Treg (**C**) cells were examined and enumerated by flow cytometry. The results were analyzed statistically with Student’s *t*-test. *: *p* < 0.05.

We further investigated the specific T cell subtypes that were affected by GMI treatment. We cocultured MDSCs and T cells for 48 h then treated the cells with anti-CD3 antibody conjugate to PerCP-Cy5.5 and anti-CD8 antibody conjugated with PE-Cy7 to label Tc cells. T_H_ cells in the T cell population were similarly labeled with anti-CD3 antibody conjugated with PerCP-Cy5 and anti-CD4 antibody conjugated with APC-H7; Treg, anti-CD3 antibody conjugate with PerCP-Cy5.5, anti-CD4 antibody conjugated with APC-H7, anti-CD25 antibody conjugated with PE, and FoxP3 conjugated with Alexa fluor 647. After flow cytometry analysis, we found that few Tc cells proliferated if the cells were untreated with GMI (Figure 3). However, when 0.5 μg GMI was added to the culture, regardless of whether MDSCs were treated or untreated with biofilms, enhancement of Tc cell proliferation became evident (Figure 3). When T cells were cocultured with MDSCs that were untreated with biofilm, 1 μg GMI increased the Tc cells by 27.5% (Figure 3). If Tc cells were cocultured with MDSCs that were treated with biofilms, a 58% increase was observed (Figure 3). GMI at 1 μg seemed to yield the maximum enhancement as 2.5 μg did not further enhance the proliferation. Unlike Tc cells, the proliferation of T_H_ cells did not increase much after treatment with GMI. When T cells were cocultured with MDSCs that were untreated with biofilms, the number of T_H_ cells increased by 4%; the proliferation increased to only 6% when they were cultured in the medium containing 1 or 2.5 μg/mL GMI (Figure 3). When T cells were incubated with MDSCs that were treated with biofilms, GMI treatment increased proliferation of T_H_ cells by 8% (Figure 3). We also found that after coculturing T cells with MDSCs or biofilm-treated MDSCs, Treg cells increased only by 0.5% and 1%, respectively (Figure 3). When the cells were cocultured in the presence of 0.5 μg GMI, GMI enhanced the proliferation of Treg cells that were cocultured with biofilm-treated MDSCs to 1.5% while the percent of proliferation was unchanged for the Treg cells when the cells were cocultured with MDSCs untreated with biofilm. In the presence of 1 or 2.5 μg GMI, proliferation of Treg increased to about 1%; when the cells were treated with biofilm, the percentages increased to 2 and 3%, respectively (Figure 3). The results showed that among the three subtypes of T cells, GMI preferentially promotes the proliferation of Tc cells that is suppressed by MDSCs.

### 3.5. Promotion of T Cell Proliferation by GMI

**Figure 4:**
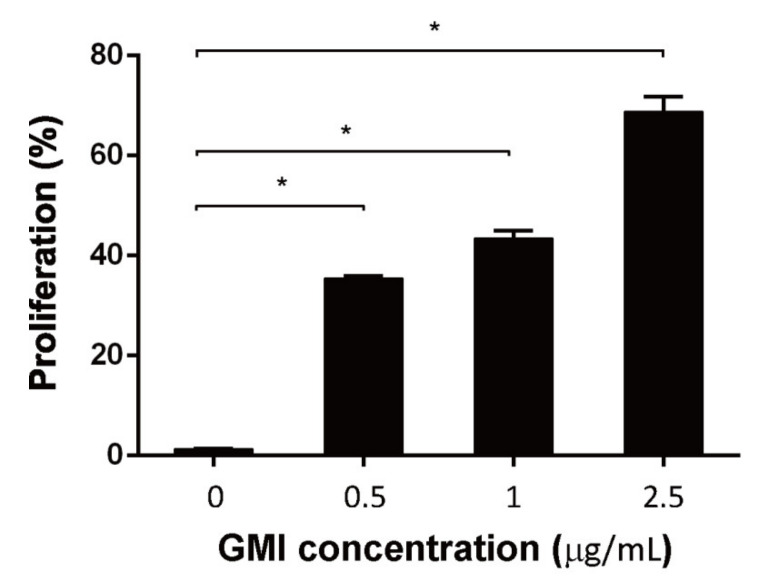
Enhancement of T cell proliferation by GMI. T cells were incubated with GMI for 48 h. T cells were then enumerated by flow cytometry. The results were analyzed statistically with Student’s *t*-test. *: *p* < 0.05.

After demonstrating that GMI promotes T cell proliferation that is suppressed by MDSCs, we further investigated whether GMI directly promoted T cell proliferation in the absence of MDSCs. We found that GMI promoted T cell proliferation in a dose-dependent manner. After adding 0.5, 1, and 2.5 μg GMI, T cell proliferation increased from 1% to 37%, 42%, and 70%, respectively (Figure 4). The results indicated that GMI can either indirectly promote T cell proliferation by reducing MDSCs’ suppression or directly enhance T cell proliferation.

### 3.6. Effects of GMI on the Production of Cytokines

**Figure 5:**
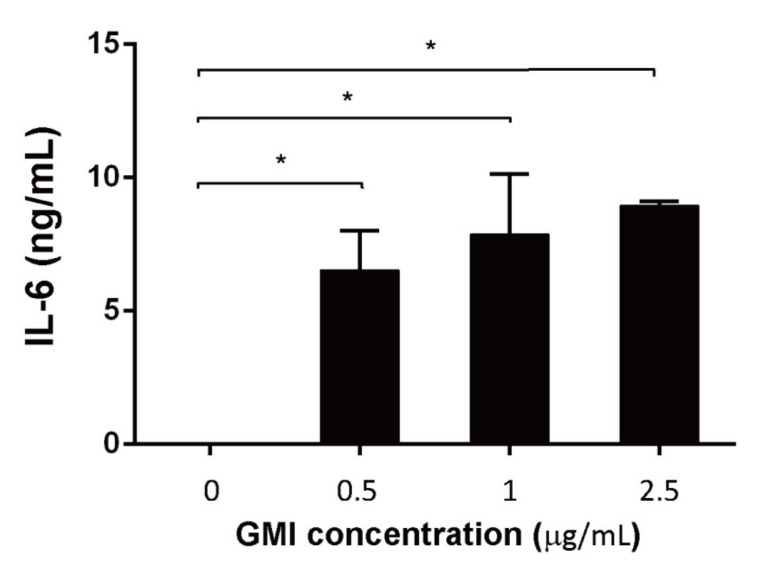
Expression of IL-6 by T cells after GMI treatment. T cells were cultured in the presence of GMI for 48 h. IL-6 in the culture medium was assayed by a CBA kit. The results were analyzed statistically with Student’s *t*-test. *: *p* < 0.05.

As it is commonly known that IL-6 promotes proliferation and activation of T cells [35], we further evaluated whether GMI treatment increased IL-6 production. After treating the cells with GMI for 48 h, IL-6 in the culture medium was captured with CBA beads and quantified by flow cytometry. We found that in the absence of GMI, little IL-6 was expressed. However, 6, 8, 9 ng/mL IL-6 were detected in the medium when cells were treated with 0.5, 1, and 2.5 μg GMI, respectively (Figure 5), showing that GMI promotes the synthesis of IL-6, from T cells.

### 3.7. Effects of GMI on S. aureus-Induced Mouse PJI Model

**Figure 6:**
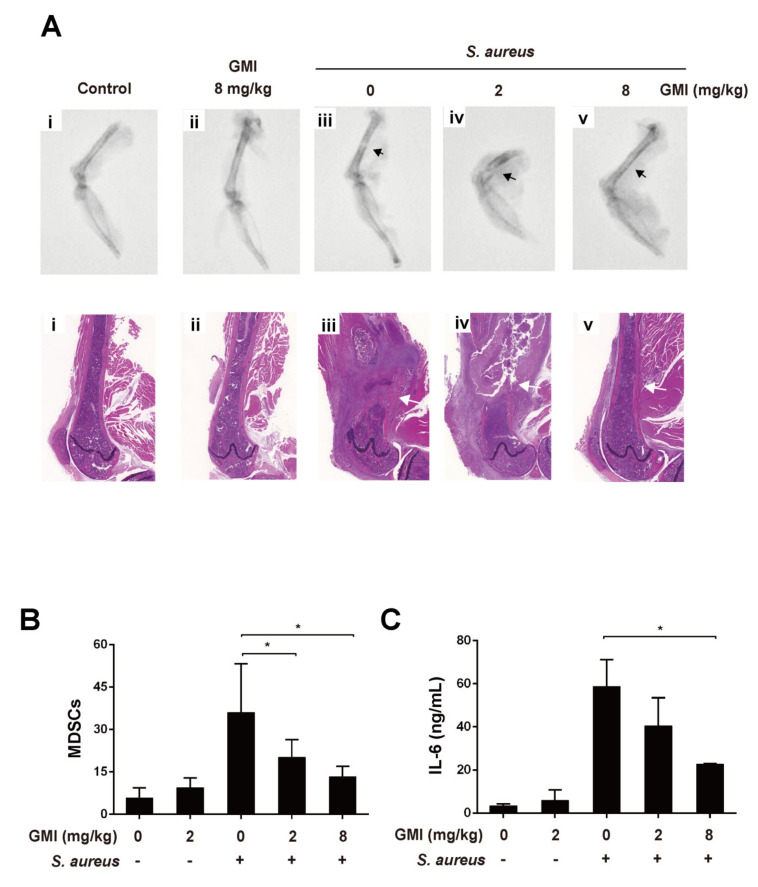
Influence of GMI treatment on *S. aureus* infection in a mouse PJI model. Mice were divided into five groups (*n* = 4): (i) control group (ii) GMI (8 mg/kg) only; (iii) infected with *S. aureus* only; (iv,v) infected with *S. aureus* and injecting 2 and 8 mg/kg GMI. (**A**) X-ray images (upper) and histological staining (lower) of femur in mice infected with *S. aureus* are shown. Black arrow indicates *S. aureus* infection site and bone destruction; White arrow showed infiltrating mononuclear immune cells. (**B**) MDSCs in PBMCs were enumerated by flow cytometry. (**C**) The concentration of IL-6 in GMI-treated mice that were uninfected or infected with *S. aureus* was determined using a CBA kit. The results were analyzed statistically with Student’s *t*-test. *: *p* < 0.05.

This study used an *S. aureus* induced mouse PJI model [20,36] to evaluate whether GMI is useful for treating PJI. As shown in Figure 6A, the X-ray image showed that the bone had severe permeated pathologic fractal after *S. aureus* infection. We also observed that treatment with 8 mg/kg GMI reduced osteolytic destructions (Figure 6). We also studied whether GMI-suppressed MDSC expansion and cytokine changes could be observed in the mouse PJI model. *S. aureus* infection increased MDSCs levels in PBMCs by 21-fold as compared to the control group (Figure 6). We found that MDSC expansion was reduced in GMI treatment groups in a dose-dependent manner; the groups treated with 2 mg/kg and 8 mg/kg GMI decreased the number of MDSCs by 30.7% and 61.9%, respectively (Figure 6B). A similar pattern was obtained for the IL-6 in blood (Figure 6C). *S. aureus* infection caused a 10.4-fold increase in IL-6. The level decreased to 41% and 60.9% after the mice were injected with 2 mg/kg GMI and 8 mg/kg GMI, respectively (Figure 6). The results showed that GMI not only reduces MDSC expansion but also increases expression of IL-6 in mice.

**Figure 7:**
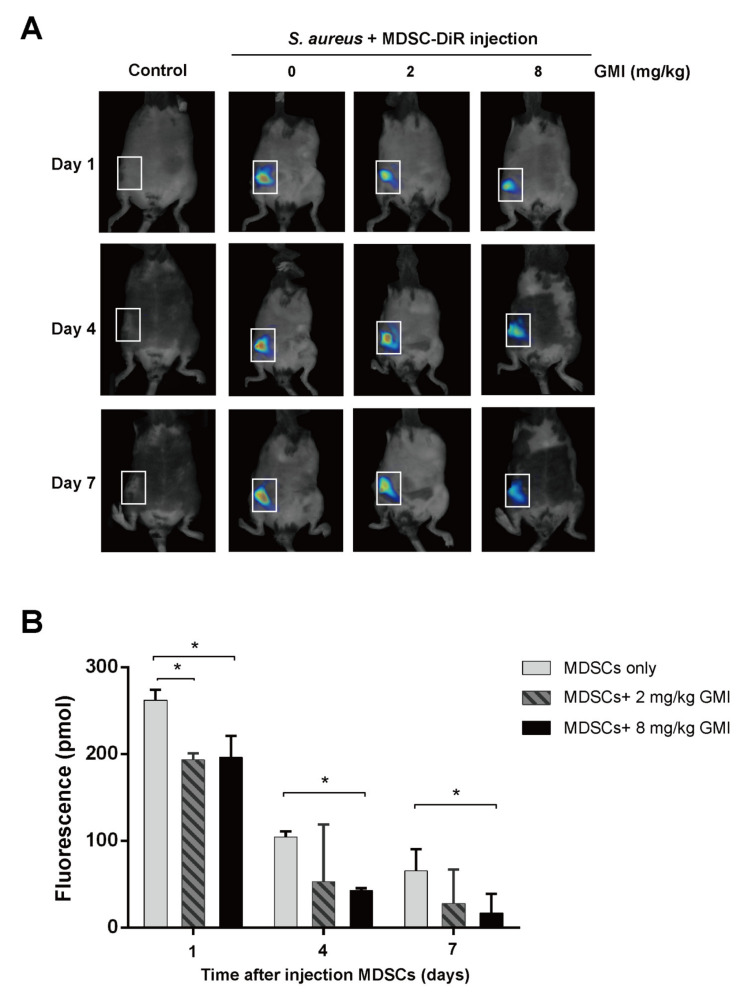
Effects of GMI on the recruitment and accumulation of local MDSCs in the infected regions after *S. aureus* infection. (**A**) The FMT images obtained on day 1, 4, and 7 after injection. (**B**) Fluorescence of MDSCs were quantitatively assessed with TrueQuan software. The results were analyzed statistically with Student’s *t*-test. *: *p* < 0.05.

It is known that *S. aureus* promotes local MDSC accumulation in the infected regions [37,38,39,40]. To demonstrate how GMI affected the accumulation of MDSC in a prosthesis, we injected mice with 5 × 10^6^ MDSCs that were labeled with a near-IR fluorescent lipophilic cyanine dye DilC18 (7) (‘DiR’) via tail veins, and the infected site was observed by fluorescence molecular tomography (FMT) at Days 1, 4, and 7. We found that introducing GMI decreased the aggregation of MDSC-DiR in the lesion region with a notably decrease during the studying period; MDSCs-DiR signals decreased in group injected with 8 mg/kg GMI compared to the control PJI group. The signal intensity decreased by 25.2%, 58.1%, and 74.3% at Days 1, 4, and 7, respectively (Figure 7). The results showed that GMI reduces the accumulation of MDSCs at the injection sites.

## 4. Discussion

PJI is a disaster after total joint arthropathy. Treatment of the disease requires surgical intervention and a prolonged antibiotic course. As the failure rate of PJI treatment is high and the disease is often associated with high morbidity and functional loss, effective treatment methods are urgently needed [6]. It is commonly known that PJI caused by *S. aureus* infection is particularly troublesome as *S. aureus* biofilms often persist on the surface of the implants and are difficult to remove [1]. Additionally, the biofilms are known to reduce host’s immunity and increase resistance to antibiotics [41], causing the treatment of *S. aureus* infection extremely difficult. This study finds that GMI is an effective agent which promotes T cell proliferation that is suppressed by *S. aureus*, making this compound potentially useful for treating PJI.

MDSC is an innate immune cell subset that decreases immune-mediated pathology of the hosts to prevent the collateral damage caused by robust host immune responses [19]. However, the decreased immunity often provides advantages to bacterial infection [19]. In an earlier study, we demonstrated that the biofilms formed by *S. aureus* strains USA300 and Col induce the expansion of MDSC, indicating that *S. aureus* exploits the expansion of MDSCs and reduced T cell proliferation to benefit their infection. We also demonstrated that curcumin inhibits MDSC expansion [36], showing that natural compounds may be useful for treating PJI. As *G. microsporum* has been widely used as a new dietary ingredient to modulate immunity, this study evaluates a peptide, GMI, isolated from this fungus, to determine whether this compound has similar effects as curcumin on inhibiting MDSC expansion. We found that GMI at 2.5 μg substantially reduces the MDSC expansion that is induced by *S. aureus* biofilms (Figure 1A,B), showing that GMI is potentially useful for treatment PJI caused by *S. aureus*.

In this study, we cocultured MDSCs and T cells to examine how GMI influences the suppression of T cell proliferation by MDSCs. We found that when coculturing T cells with MDSCs, a 30% T proliferation was observed; a 20% proliferation was observed when T cells were cocultured with MDSCs from BMCs treated with *S. aureus* biofilms (Figure 2). This finding is consistent with results that *S. aureus* biofilms stimulate MDSC expansion to inhibit T cell proliferation [20]. This study also showed that GMI promoted T cell proliferation that is suppressed by both types of MDSCs. At 2.5 μg/mL of GMI, the suppression of T cell proliferation by MDSCs and the MDSCs from biofilm-treated BMCs was reduced and a 60% proliferation was observed (Figure 2), showing that GMI significantly reduced the suppression of T cell proliferation by MDSCs from biofilm-treated BMCs. More importantly, Tc cells were more responsive to GMI treatment than T_H_ and Treg cells, showing that GMI specifically reduced the suppression of Tc proliferation by MDSCs. The stimulation of T cell proliferation by GMI may be attributed to its ability to promote the expression of IL-6 and TNF-α (Figure 1). GMI is an FIP with a sequence and structure similar to Lingzhi 8 (LZ8) from *Ganoderma microsporum*. LZ-8 is known to induce mouse splenocytes, human peripheral blood lymphocytes, PBMC, and T cell proliferation and activation [22,24,42]. LZ-8 also upregulates the expression of IL-2, TNF-α, IFN-γ, and IL-1β [43,44]. It is well known that IL-6, TNF-α, and IL-1, influence MDSC expansion [40,45,46] and promote the T cell proliferation [35]. Meanwhile, the expression of IL-6 reduces apoptosis of CD4 T cells [47], increases activated T cells migration [48], and regulates Th1/Th2 balance toward Th2 differentiation [49]. Importantly, IL-6 promotes differentiation of naïve CD8 T cell into a unique population of effector CD8+ T cells for antagonizing the viral infection [50]. It is also known that TNF-α promotes T cell activation and proliferation [51]. The increases in TNF-α and IL-6 levels may explain how GMI promotes proliferation T_c_ cells.

Finally, we examined how the administration of GMI benefits the treatment of PJI in an *S. aureus*-induced PJI mouse model. We found that GMI treatment reduced osteolytic destructions (Figure 6). We also found that MDSC expansion was reduced in GMI treatment groups but increased IL-6 and TNF-α expression, which were consistent with the in vitro results (Figure 1, Figure 2, Figure 3 and Figure 4). It is known that *S. aureus* promotes local MDSC accumulation in the infected regions [37,38,39,40]. To demonstrate how GMI affected the accumulation of MDSC in prosthesis, we injected mice MDSCs and observed by fluorescence molecular tomography (FMT), the aggregation of MDSC in the lesion reduced significantly during the studying period, showing that GMI reduces accumulation of MDSCs at the injection sites. This study demonstrates that GMI is an attractive compound potentially beneficial to the treatment of PJI.

## Data Availability

Not applicable.
